# Metabolomic change due to combined treatment with myo-inositol, D-chiro-inositol and glucomannan in polycystic ovarian syndrome patients: a pilot study

**DOI:** 10.1186/s13048-019-0500-x

**Published:** 2019-03-23

**Authors:** Jacopo Troisi, Claudia Cinque, Luigi Giugliano, Steven Symes, Sean Richards, David Adair, Pierpaolo Cavallo, Laura Sarno, Giovanni Scala, Maria Caiazza, Maurizio Guida

**Affiliations:** 1 Department of Medicine, Surgery and Dentistry, “Scuola Medica Salernitana”, University of Salerno, Baronissi, SA Italy; 2 Theoreo srl, Via degli Ulivi 3, 84090 Montecorvino Pugliano, SA Italy; 3 European Biomedical Research Institute of Salerno (EBRIS), Via S. de Renzi, 3, 84125 Salerno, SA Italy; 4 Department of Chemistry and Physics, University of Tennessee at Chattanooga, 615 McCallie Ave., Chattanooga, TN 37403 USA; 5 Department of Obstetrics and Gynecology, University of Tennessee College of Medicine, Chattanooga, TN USA; 6 Department of Biology, Geology and Environmental Sciences, University of Tennessee at Chattanooga, 615 McCallie Ave., Chattanooga, TN 37403 USA; 7 Department of Physics, University of Salerno, Fisciano, SA Italy; 8 Department of Neurosciences and Reproductive and Dentistry Sciences, University of Naples Federico II, Naples, Italy; 9 Hosmotic srl, Via Raffale Bosco 78, 80069 Vico Equense, NA Italy; 10 Azienda Sanitaria Locale, distretto sanitario 66, via Vernieri, 14, 84124 Salerno, SA Italy; 11 Istituto Sistemi Complessi – Consiglio Nazionale delle Ricerche, Rome, Italy

**Keywords:** Polycystic ovary syndrome, Inositols-glucomannan association, Metabolomics

## Abstract

**Background:**

Polycystic ovarian syndrome (PCOS) is a highly variable syndrome and one of the most common female endocrine disorders. Although the association inositols-glucomannan may represent a good therapeutic strategy in the treatment of PCOS women with insulin resistance, the effect of inositols on the metabolomic profile of these women has not been described yet.

**Results:**

Fifteen PCOS-patients and 15 controls were enrolled. Patients were treated with myo-inositol (1.75 g/day), D-chiro-inositol (0.25 g/day) and glucomannan (4 g/day) for 3 months. Blood concentrations of glucose, insulin, triglycerides and cholesterol, and ovary volumes and antral follicles count, as well as metabolomic profiles, were evaluated for control subjects and for cases before and after treatment.

PCOS-patients had higher BMI compared with Controls, BMI decreased significantly after 3 months of treatment although it remained significantly higher compared to controls. 3-methyl-1-hydroxybutyl-thiamine-diphosphate, valine, phenylalanine, ketoisocapric, linoleic, lactic, glyceric, citric and palmitic acid, glucose, glutamine, creatinine, arginine, choline and tocopherol emerged as the most relevant metabolites for distinguishing cases from controls.

**Conclusion:**

Our pilot study has identified a complex network of serum molecules that appear to be correlated with PCOS, and with a combined treatment with inositols and glucomannan.

**Trial registration:**

ClinicalTial.gov, NCT03608813. Registered 1st August 2018 - Retrospectively registered, .

**Electronic supplementary material:**

The online version of this article (10.1186/s13048-019-0500-x) contains supplementary material, which is available to authorized users.

## Background

Polycystic ovary syndrome (PCOS) is a heterogeneous syndrome and one of the most common female endocrine disorders, affecting 5–20% of women in reproductive age [1, 2]. Clinical expression is highly variable, but typically includes oligo-ovulation or anovulation, hyperandrogenism and polycystic ovaries [3]. PCOS is associated with an increased risk of type 2 diabetes, cardiovascular events [4] and endometrial cancer [5]. Insulin Resistance (IR) plays a central role in approximately 70–80% of obese women and in 15–30% of lean women with PCOS [3], and represents the pathogenic link between metabolic and reproductive disorders in PCOS [6].

The last decade has seen the introduction of inositols for the treatment of PCOS and a recent review suggests that inositol therapy can reduce insulin resistance, improve ovarian function, and reduce androgen levels in women with PCOS [7]. The two inositol isomers of interest are myo-inositol (MI) and D-chiro-inositol (DCI) which often have a physiological ratio of 40:1 in most healthy tissues [8]. Importantly, the MI:DCI ratio does vary between healthy and disease state [9–11] thus strengthening the hypothesis that inositol therapy may be beneficial for maintaining metabolic, endocrine, and reproductive health in women with PCOS.

Glucomannan is a water-soluble fiber that is derived from the konjac root [12]. It has been reported as being able to suppress hepatic cholesterol synthesis and increase the elimination of cholesterol-containing bile acids [13]. Some authors have suggested that glucomannan ingestion could also increase the gastric emptying time [14–16]. This effect may be related to the increased feeling of satiety reported in some studies [17]. The European Food and Safety Authority (EFSA) in 2010 reported a scientific opinion supporting the anti-cholesterol and weight reduction claims for glucomannan-containing drugs [18].

The association inositols-glucomannan may represent a good therapeutic strategy in the treatment of PCOS women with insulin resistance [19].

The use of metabolomics for the study of disease mechanisms is increasing. Metabolites are low-molecular-weight organic and inorganic chemicals that are the substrates, intermediates, and byproducts of enzyme-mediated biochemical reactions in the cell [20]. Several thousand metabolites in human serum have been identified so far, and the application of metabolomics has allowed the development of biomarkers which provides new insight for many diseases [21].

Metabolomic analysis in gynecology has been extended to studies of various malignancies, such as ovarian cancer [22] and endometrial cancer [23, 24]. Moreover, many studies have incorporated a metabolomic approach to better define the pathophysiology of PCOS [25–31] and to describe how different therapies can modify metabolic profiles [32–34].

However, the effect of inositols on the metabolomic profile of women with a diagnosis of PCOS has not been described yet. Thus, the aim of this study is to analyze the metabolic profiles in women with PCOS before and after 3 months of therapy with a combination of MI, DCI and glucomannan, and compare these data with a group of healthy control women.

## Results

Based on serum metabolomic signatures of PCOS patients and controls a statistical power of 80% resulted from the analysis of at least 15 subjects for each class (See Additional file 1: Figure S1). The demographic and clinical–biochemical characteristics of PCOS patients at baseline (PCOS-T0) and after 3 treatment months (PCOS-T1) and controls (CTRL) are reported in Table 1. All PCOS patients met both the Rotterdam ESHRE/ASRM-Sponsored PCOS Consensus Workshop Group Criteria [4] and the adolescent criteria [35] for the diagnosis of PCOS. All subjects in this study were unmarried, not interested in soon becoming pregnant, and were not currently taking hormonal contraceptives. None of them had previously given birth or had undergone voluntary interruption of pregnancy. None of the controls displayed any biochemical or gynecological abnormalities. Women with PCOS had higher Body Mass Index (BMI) compared with Controls (mean ± standard deviation, 28.4 ± 1.7 vs. 21.6 ± 1.6 [kg/m^2^], *p* < 0.05) and BMI decreased significantly (*p* < 0.05) after 3 months of treatment even if it remained significantly higher compared to controls (26.4 ± 1.7 vs. 21.6 ± 1.6 [kg/m^2^], *p* < 0.05). Average blood Glucose, Insulin and Homeostatic Model Assessment for Insulin Resistance (HOMA-IR) values were not significantly different, although the mean values for each parameter were greater in PCOS samples. These values did not significantly change after 3 months of treatment. Menstrual cycle regularity improved significantly after treatment. The number of antral follicles and the ovary volume were higher in PCOS patients (6.8 ± 1.2 vs. 15.4 ± 1.7, *p* < 0.05 and 7.4 ± 0.7 vs. 12.2 ± 2.2 [mL], *p* < 0.05, respectively) but both decreased after treatment (13.2 ± 2.3 and 10.2 ± 1.9 [mL], *p* < 0.05, respectively). The same trend was observed for the Ferriman-Galleway and the acne score (see Table 1).

**Table 1:** Anamnestic and anthropometric characteristics of controls and cases at enrollment (PCOS-T0) and after 3-months treatment (PCOS-T1)

	Control (*n* = 15)	PCOS-T0 (*n* = 15)	PCOS -T1 (*n* = 15)	Reference values
Age (years)	21.9 ± 2.9	19.7 ± 1.9		–
Height (cm)	158.0 ± 6.4	159.2 ± 7.2		–
Weight (Kg)	53.9 ± 6.0	71.9 ± 6.7*	67.0 ± 7.3*	–
BMI (Kg/m^2^)	21.6 ± 1.6	28.4 ± 1.7*	26.4 ± 1.7^*,§^	< 18.5 Underweight 18.5–25.0 Normal weight 25.1–29.9 Overweight > 30 Obese
Menstrual flux				
Normal	12 (80%)	5 (33%)	9 (60%)	< 20 mL
hypo-	2 (13%)	9 (60%)	5 (33%)	20–80 mL
hyper-	1 (7%)	1 (7%)	1 (10%)	> 80 mL
Blood Glucose (mg/dL)	79.3 ± 6.9	83.9 ± 7.5	88.0 ± 8.5*	70–110
Insulin (μU/mL)	6.2 ± 1.2	8.0 ± 3.1	7.9 ± 3.8	2–25
HOMA-IR index	1.30 ± 0.22	1.68 ± 0.64	1.76 ± 0.99	0.2–2.2
Total Cholesterol (mg/dL)	150.7 ± 17.3	169.7 ± 34.9	152.1 ± 21.5	< 200
Triglycerides (mg/dL)	67.0 ± 17.1	77.6 ± 26.0	62.5 ± 12.3	< 150
Total Testosterone (ng/dL)	32.7 ± 9.88	42.7 ± 9.91*	34.98 ± 8.11	21–49
Free Testosterone (pg/mL)	2.49 ± 0.81	2.50 ± 0.98	2.48 ± 1.06	< 3.0
Menstrual cycle regularity				
Yes	15 (100%)	0 (0%)*	4 (27%)*	Regularity: loss between 20–80 mL; cycle lasting 25–36 days
No	0 (0%)	15 (100%)*	11 (73%)*	
Number of menstrual cycles in previous 3 months	3.0 ± 0.0	1.6 ± 0.5*	2.0 ± 0.7*	
Number of antral follicles	6.8 ± 1.2	15.4 ± 1.7*	13.2 ± 2.3^*,§^	< 12
Acne score	1.4 ± 0.5	2.1 ± 1.2	1.8 ± 1.1	–
Ovary volumes (mL)	7.4 ± 0.7	12.2 ± 2.2*	10.2 ± 1.9^*,§^	< 10
Ferriman-Gallwey score	5.1 ± 2.1	15.5 ± 5.0*	15.0 ± 4.9*	< 8

Patient characteristics are reported as mean ± standard deviation or number (percentage). * *p*-value < 0.05 PCOS-T0 or PCOS-T1 vs Control, ^§^*p*-value < 0.05 PCOS-T1*vs* PCOS-T0

**Fig. 1:**
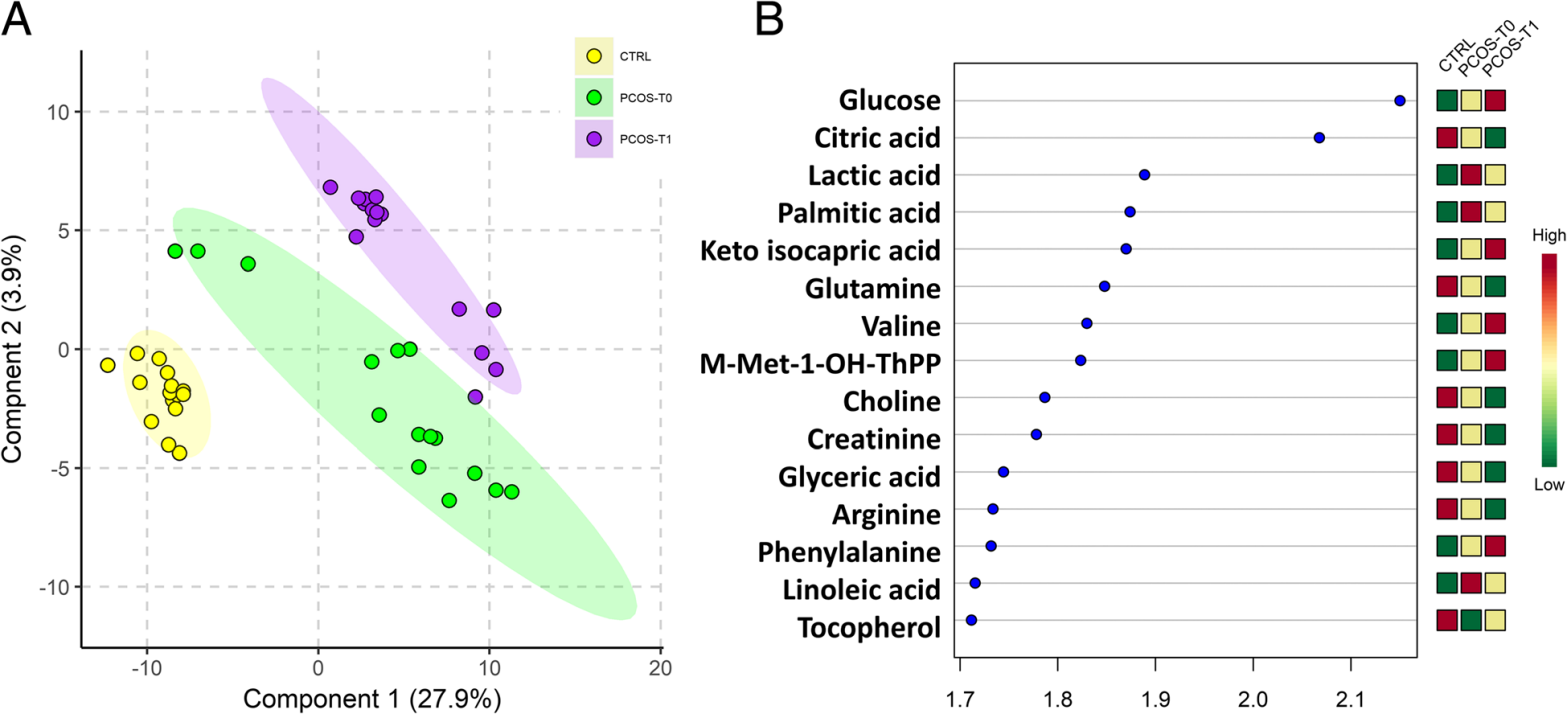
**a** Partial least square discriminant analysis (PLS-DA) models to discriminate Controls (CTRL, yellow circles), PCOS patients at enrolment (PCOS-T0, green circles) and PCOS patients after 3-months treatment (PCOS-T1, purple circles). The explained variance of each component is shown in parentheses on the corresponding axis. **b** The 15 top-scoring VIP metabolites (VIP-score ≥ 1.5) are shown. The colored boxes on the right indicate the relative amount of the corresponding metabolite in each group under study

As shown in Fig. 1a, the partial least square-discriminant analysis (PLS-DA) score plots clearly differentiated three groups consisting of controls, PCOS patients before treatment, and PCOS patients after treatment. The 15 highest scoring VIP variables (VIP score > 1.5) identified by PLS-DA are shown in Fig. 1b. The metabolite concentration changes induced by the therapy allowed division of the metabolites into three classes: those with higher concentrations than the controls and which increased further after therapy, those which have higher concentrations than the controls and whose concentration decreased after therapy and, those with lower concentrations compared to controls that did not change after therapy.

**Fig. 2:**
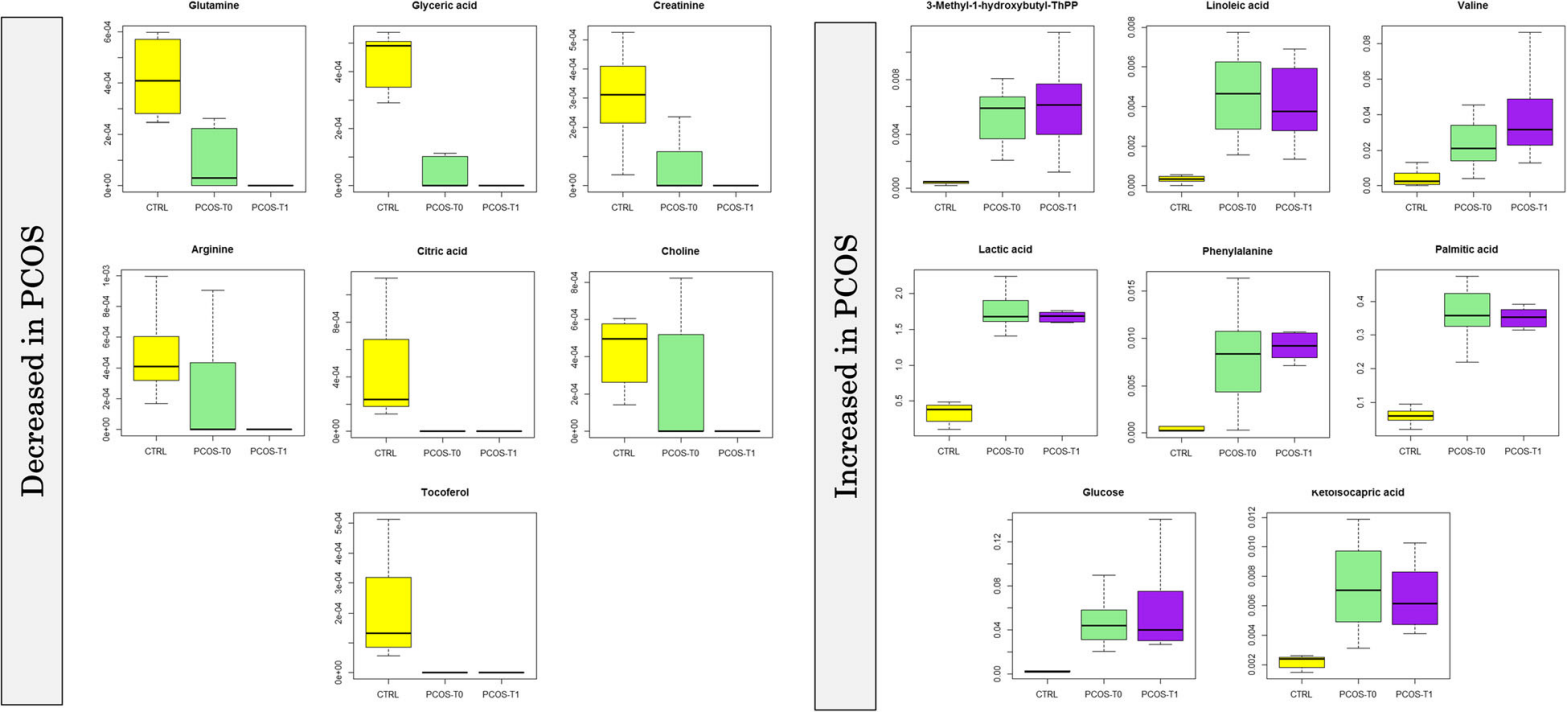
Box and Whisker plot of the VIP metabolites in the cohort of patients and controls. Boxes represent controls (CTRL) *n* = 15; PCOS patients at the basal enrolment time (PCOS-T0), *n* = 15; and PCOS patients after 3-months treatment (PCOS-T1), *n* = 15). The vertical axis reports the log of the GC-MS value of the normalized area of each metabolite. Abbreviations: Gas Chromatography-Mass Spectrometry (GC-MS), Polycystic Ovary Syndrome (PCOS), Thiamine Phosphate (ThPP)

As shown in Fig. 2, the concentrations of 3-methyl-1-hydroxybutyl-thiamine diphosphate (FC = 6.94, *p* < 0.001), valine (FC = 4.87, *p* = 0.001) and phenylalanine (FC = 10.91, *p* < 0.001) were higher in PCOS-T0 patients compared to the control and increased even further after the treatments (FC = 1.13, *p* = 0.001; FC = 1.71, *p* = 0.002; FC = 1.17, *p* < 0.001, comparing PCOS-T1 to PCOS-T0, respectively).

Ketoisocapric acid (FC = 3.12, *p* < 0.001), linoleic acid (FC = 14.76, *p* < 0.001), lactic acid (FC = 5.31, *p* < 0.001), palmitic acid (FC = 6.22, *p* < 0.001) and glucose (FC = 19.92, *p* < 0.001), were also higher in PCOS patients at enrollment time but they decreased after treatments (FC = 0.93, *p* < 0.001; FC = 0.90, *p* < 0.001; FC = 0.93, *p* < 0.001; FC = 0.93, *p* < 0.001; FC = 0.89, *p* = 0.001, respectively).

Glutamine (FC = 0.41, *p* > 0.05), glyceric acid (FC = 0.85, *p* > 0.05), creatinine (FC = 0.71, *p* > 0.05), arginine (FC = 0.50, *p* > 0.05), citric acid (FC = 0.45, *p* > 0.05), choline (FC = 0.55, *p* > 0.05) and tocopherol (FC = 0.59, *p* > 0.05) were all lower in PCOS patients and had very low concentrations after treatment although these values were not significantly different.

**Fig. 3:**
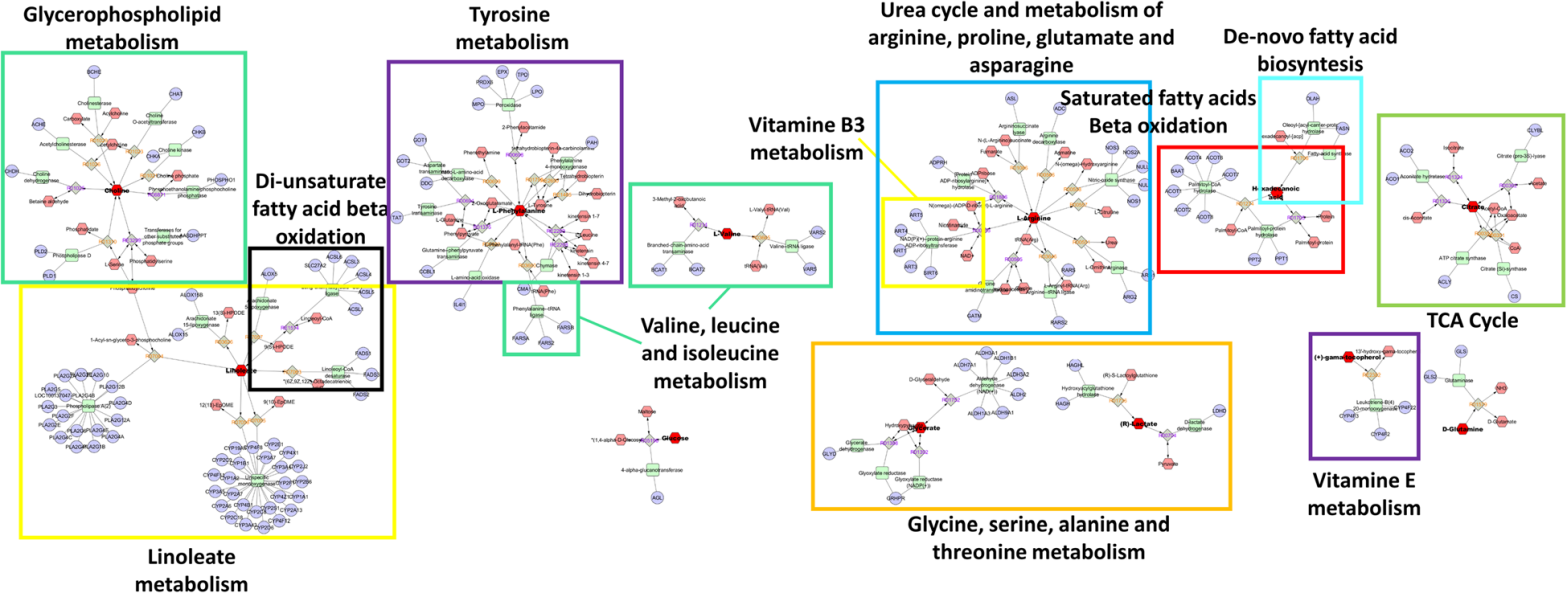
Metabolic systems map summarizing the shortest route that may explain the interactions among the 15 selected metabolites. There is a clear interplay of several pathways involving: Biopterin metabolism; De novo fatty acid biosynthesis; Di-unsaturated fatty acid beta-oxidation; Glycerophospholipid metabolism; Glycine, serine, alanine and threonine metabolism; Linoleate metabolism; Saturated fatty acids beta-oxidation; TCA cycle; Tyrosine metabolism; Urea cycle and metabolism of arginine, proline, glutamate, aspartate and asparagine; Valine, leucine and isoleucine degradation; Vitamin B3 (nicotinate and nicotinamide) metabolism; Vitamin E metabolism

The metabolic pathway analysis of the selected metabolites is summarized in the metabolic systems map shown in Fig. 3. There is a definite interaction of several pathways involving Biopterin metabolism; De novo fatty acid biosynthesis; Di-unsaturated fatty acid beta-oxidation; Glycerophospholipid metabolism; Glycine, serine, alanine and threonine metabolism; Linoleate metabolism; Saturated fatty acids beta-oxidation; TCA cycle; Tyrosine metabolism; Urea cycle and metabolism of arginine, proline, glutamate, aspartate and asparagine; Valine, leucine and isoleucine degradation; Vitamin B3 (nicotinate and nicotinamide) metabolism; Vitamin E metabolism.

## Discussion

This is a prospective case-control study conducted in a single center at University Hospital “San Giovanni di Dio e Ruggi D’Aragona” of Salerno.

The studied population included only young/adolescent women. We have chosen to investigate this specific age group, since younger women may benefit the most from an early treatment in terms of future pregnancy chances and possibly avoiding comorbidities development [36]. Furthermore, the PCOS-inositols treatments do not contraindicate other concomitant therapeutic options [7].

The aim of this study was, firstly, to identify discriminant metabolites in serum of women with a diagnosis of PCOS and, secondly, to define if treatment with inositols can improve metabolomic pathways and metabolic profiles of women with PCOS. Multi-variate statistical analysis identified fifteen metabolites as being particularly important in separating cases from controls: 3-methyl-1-hydroxybutyl-thiamine diphosphate, valine, phenylalanine, ketoisocapric acid, linoleic acid, lactic acid, palmitic acid and glucose were increased in PCOS patients compared to controls, while glutamine, glyceric acid, creatinine, arginine, citric acid, choline and tocopherol were decreased.

According to these results, different metabolic pathways appear to be involved in PCOS pathology. In the serum of PCOS patients at enrollment time in comparison with control group, several metabolites, closely associated with carbohydrate and lipid metabolisms, are significantly dysregulated.

Increased levels of lactic acid have been previously reported in PCOS patients [27, 37] and can be related to an increased muscular gluconeogenesis or can indicate an uptake and consumption of glucose by muscles, suggesting an important role for insulin resistance in these patients [25].

As previously reported [27], linoleic acid and palmitic acid are also increased, likely the result of increased lipolysis that might be related to presence of insulin resistance in fatty tissue [25]. PCOS is associated with increased lipolysis due to presence of IR in adipose tissue [38]. However, IR does not occur in all tissues of all women with PCOS, and different mechanisms may contribute to hyperinsulinemia [39].

Moreover, high abundances of linoleic acid appear to inhibit the maturation and development of oocytes [40]. Due to proinflammatory activity, elevated linoleic acid levels can lead to the chronic inflammation associated with PCOS [41, 42].

Several amino acids and other metabolites associated with the tricarboxylic acid cycle (TCA) are altered in PCOS patients: the levels of arginine, choline, glutamine and citric acid were decreased and those of phenylalanine and valine were increased, compared to the controls. Such alterations to the TCA cycle are also related to insulin resistance [43] and alterations in oocyte maturation [44].

Valine was increased in PCOS patients as previously reported [29] and it might be associated with other metabolic alterations not related with insulin resistance [25]. As with the present study, creatinine has been reported to be reduced in PCOS patients [37].

Nevertheless, our specific cohort showed a low incidence of insulin resistance, according to previous reported data on adolescent population [35, 45], therefore the proposed theories/explanation may not fully apply to the cohort of patients evaluated in this study.

We found a reduction of tocopherol in PCOS patients, consistent with previous work [39]. This metabolite has an anti-oxidant effect and the reduction in concentration might be explained by the increased oxidative stress observed in PCOS.

According to recent guidelines, insulin-sensitizer drugs are the first-line therapy in women with metabolic abnormalities and irregular menstrual cycle with the purpose to improve fertility, whereas a lifestyle change with weight loss and physical activity is the first step in overweight and obese PCOS patients [46]. Inositols belong to a sugar alcohol family comprising nine stereoisomers and two of them, MI and DCI, are involved in the pathophysiology and treatment of PCOS [47]. Inositols work as second messengers of insulin: MI improves oocyte quality mediating glucose uptake and follicle stimulating hormone (FSH) signaling, while the supplementation of DCI, is involved in insulin-mediated androgen synthesis, replenishing stores of the mediator and improving insulin sensitivity [7, 47, 48]. A recent review has shown the efficacy of inositols treatment in the management of PCOS [49], while an Italian study [19] has shown the synergistic action of inositols-glucomannan in the treatment of PCOS women with insulin resistance (IR). So, we can suggest a central but not peripheral role of IR in the present cohort. In another study, after combined treatment with inositols-glucomannan, PCOS patient showed decreased levels of free fatty acids, and patients experienced weight reduction [50]. In addition, the reduction of linoleic acid was also reported after 30 months of polytherapy with pioglitazone-flutamide-metformin and hormonal medication in an adolescent patient group with PCOS [32]. Moreover, a recent study [33] has shown how, in metabolic terms, 3-iodothyronamine administration in a mouse model of PCOS may serve as a promising endogenous supplement in the treatment of dysfunctional lipid metabolism.

It would be reasonable to expect a trend towards normalization of the metabolome upon weight reduction and clinical improvement due to the treatment. Indeed, after 3 months of treatment with a combination of MI, DCI and glucomannan, the clinical symptoms did improve. However, the metabolic profiles of patients after 3 months of treatment were less close to those of controls compared to initial metabolomes. Indeed, metabolites with lower abundances in PCOS-T0 patients either stayed the same or decreased even more after treatment, while those metabolites with higher abundances generally increased after treatment. We offer two potential explanations for these observations: 1) the metabolome is more strongly affected by therapeutically induced rapid weight loss (approximately 400 g/week) in overweight patients compared to PCOS condition; 2) the metabolome of patients with PCOS may adapt to this condition, thus rendering the differences between controls and not-treated patients less prominent. This effect was observed in anorexic women treated with a food therapy [51]. Similarly, it can be argued that the strongly altered metabolome of weight loss patients would normalize if the achieved body weight is maintained. In general terms, we conclude that metabolic processes are seemingly susceptible to changes generated by being overweight and even more so by subsequent weight loss. The modest improvement in menstrual cycle regularity following treatment (Table 1) is statistically consistent with that reported by Pundir’s meta-analysis [52]. This can be related to the lower incidence of IR in our cohort; indeed a large amount of evidence indicates that lowering insulin levels with insulin-sensitizing drugs in IR-PCOS women can reduce circulating androgen levels, increase SHBG levels, and restore ovulatory menstrual cycles [53]. Moreover, although only 27% of the post-treatment subjects reported menstrual cycle characteristics consistent with a “regular” definition (i.e., a cycle lasting between 25 and 36 days and menstrual blood loss between 20 and 80 mL), all the patients reported some kind of improvement in menstrual cycle regularity.

Our study has a few limitations. The first is the lack of a metabolomic comparison among PCOS patients undergoing inositols-only treatment. As a result, we cannot address which changes were due to the combined treatment and which ones were due to inositols. A further limitation is the small number of patients enrolled, although acceptable statistical power [54] is still achieved. Our PCOS patients did not show higher free and total testosterone levels compared with reference values of the Italian population. However, many factors are considered for diagnosis of hyperandrogenism. The Androgen Excess Society criteria for diagnosis of PCOS requires clinical *or* biochemical evidence of hyperandrogenism [55].

*Clinical* diagnosis of hyperandrogenism is identified by hirsutism (as scored by the Ferriman-Gallwey exam), acne, and alopecia [56]. *Biochemical* diagnosis of hyperandrogenism includes measuring serum total testosterone, free testosterone, free androgen index, androstenedione, and dehydroepiandrosterone sulfate [57]. Amirie et al. [58], conducted a meta-analysis of 6593 PCOS patients and confirmed that the Ferriman-Gallwey *clinical* diagnostic criteria significantly correlates with *biochemical* diagnostic criteria. Concentrations of androstenedione and dehydroepiandrosterone sulfate correlated with the Ferriman-Gallwey diagnosis of PCOS; while, as with our study, a significant correlation was not found with testosterone and free testosterone [58]. Indeed, the most commonly quantified biochemical parameters of hyperandrogenism are free testosterone and free androgen index; however, the increased levels of androstenedione or other androgen precursors are, for many patients, the only indicators of hyperandrogenism [55, 58, 59]. Thus, the fact that free testosterone and total testosterone was not higher in our PCOS cases does not indicate the patient did not have PCOS. Therefore, all of our PCOS cases met the *clinical* criteria for hyperandrogenism, which is considered the cornerstone of PCOS [55]. Finally, our PCOS cases were also well defined by ultrasonography and Acne scores.

Despite the limitations, the present pilot study allowed us to follow the patients over a 3-month treatment regimen. Indeed, our pilot study is an ideal model because it allows metabolomic analysis of pre- and post-treatment serum taken from the same patient. This in turn allows comparative analysis of metabolite profiles from subjects for which the genetic, hormonal and environmental backgrounds have remained constant. In this way, changes in metabolome occurring during the treatment can be traced independently from the physiological inter-variability among people.

Large functional and prospective studies, including clinical trials, are needed to verify our preliminary results and to separate the role of inositols and of the combined treatment with inositol and glucomannan. Such studies will continue to shed further insights into these metabolite profiles and determine whether some of them are biomarkers or actual mediators of PCOS. A better understanding of the role of these biomarkers in obesity and obesity-related PCOS pathogenesis might finally lead to novel targets for therapeutic/preventive intervention. Furthermore, this kind of study can contribute to understanding the role of weight reduction in PCOS patients.

## Conclusions

In summary, our pilot study has identified a complex network of serum molecules that appear to be correlated with PCOS, and with a combined treatment with inositols and glucomannan. Future studies with larger samples sizes involving PCOS patients identified through clinical *and* biochemical criteria are needed to further validate our findings.

## Methods

### Population and study design

The study was designed as a prospective case-control conducted at the University of Salerno from September 2016 to July 2017. Women with a diagnosis of PCOS (according to the European Society of Human Reproduction and Embryology [4] and to adolescent PCOS diagnostic criteria [35]) were recruited, along with matched healthy controls.

Both cases and controls were enrolled at the University Hospital “San Giovanni di Dio e Ruggi d’Aragona” of Salerno, during routine visits to the gynecological clinic, whenever inclusion criteria were present. Inclusion criteria for the case group were: age between 18 and 35 years, overweight/obesity (BMI > 25 kg/m^2^), a positive diagnosis of PCOS, and absence of any other acute intercurrent or chronic illness. Healthy controls were patients between 18 and 35 years old without acute intercurrent or chronic illness.

Women using hormonal medications or drugs that affect insulin sensitivity (e.g., inositols or metformin) before enrollment were excluded.

An informed consent form was signed by each participant at enrollment. The study was carried out in accordance with the ethical principles of the declaration of Helsinki and approved by the ethics committee CEI “Comitato etico Campania Sud” (IRB N. 78/2016).

Subjects were recruited in the framework of a multicentric registered study. The study was registered in the clinicaltrial.gov portal on August 2018 with the number: NCT03608813. Only patients recruited in the Salerno hospital were used for this pilot study.

### Data collection and pharmacological treatment

At enrollment, anamnestic and demographic characteristics were collected. Specific data collected include: BMI, frequency and characteristics of menstrual cycle.

To evaluate the degree of hirsutism, the modified score of Ferriman-Gallwey [60] was used. It gives a score from 0 (no growth of terminal area) to 4 (extensive hair growth) for each of the nine body areas. An overall value of Ferriman-Gallwey score greater than, or equal to, 8 was considered as indicative of hirsutism according to the American Association of Clinical Endocrinologists Medical Guidelines for Clinical Practice for the Diagnosis and Treatment of Hyperandrogenic Disorders [61].

The degree of acne was evaluated according to the Global evaluation scale proposed in 2002 by FDA [62]. In clinical practice, menstrual cycle is considered normal when it lasts between 25 and 36 days (28 on average) with a blood loss between 28 and 80 ml; menstrual loss of modest size (< 20 ml) is considered hypomenorrhea, while, abundant menstrual loss (> 80 ml) is characteristic of hypermenorrhea condition [63]. To evaluate menstrual loss, we used the menstrual pictogram proposed by Magnay et al. [64].

Blood concentration of glucose, insulin, triglycerides and cholesterol were evaluated for control subjects and for cases at baseline (PCOS-T0) and after 3 months of treatment (PCOS-T1). HOMA-IR was also calculated. Ovary volumes and the antral follicles count were evaluated by a vaginal ultrasound performed by a trained gynecologist.

Enrolled cases were treated with myo-inositol 1.75 g/day, D-chiro-inositol 0.25 g/day and glucomannan 4 g/day using the Realim (AG Pharma, Rome, Italy) formulation. The drugs were divided into two equal parts and taken before the two principal meals for three consecutive months. After this time, the patients were re-evaluated regarding the anthropometric, biochemical and ultrasound parameters. Both groups were recommended to not change any life standards during the study period, except for the use of medications.

### Ultrasonographic evaluation

All patients underwent transvaginal ultrasonography (US) performed by four experienced gynecologists (C.C., M.C., L.G. and M.G.) using a MyLab Seven ultrasound machine (Esaote, Genova, Italy) with a 5 MHz convex probe using a transvaginal approach. This approach made it possible to accurately evaluate the internal structure, even in obese patients, in whom the trans-abdominal scan was not sufficiently reliable. Transvaginal ultrasonography was performed with the woman in the lithotomy position and empty bladder. To correctly visualize the annexes, we moved the probe to the right for the right adnexal region and to the left for the left adnexal region; furthermore, by rotating the probe and making movements in the anterior-posterior direction, both on the right and on the left, we obtained oblique-longitudinal scans that allowed us to evaluate the entire adnexal region. The position, the echostructure, the size of the ovaries and the number of follicles for each of them were evaluated. In particular, for the dimensions, we measured three orthogonal diameters for each ovary (defined D1, D2 and D3) and then the US machine software applied the ellipsoid formula: π/6 X (D1 X D2 X D3). For the number of follicles, the calculation included all the follicles present on each ovary, from margin more internal to the outermost one, regardless of their arrangement, and, for a more exhaustive study. According to the Rotterdam ESHRE/ASRM-Sponsored PCOS Consensus Workshop Group, presence of 12 or more follicles in each ovary, measuring 2–9 mm in diameter, and/or increased ovarian volume (> 10 mL) are the criteria with sufficient specificity and sensitivity for the diagnosis of PCOS.

### Samples collection

Human tissue collection strictly adhered to the guidelines outlined in the Declaration of Helsinki IV edition [65]. Blood samples were collected at enrollment from controls and at enrollment and three months after the treatment for cases, using a BD vacutainer (Becton Dickinson, Oxfordshire, UK) blood collection red tube (with no additives). After centrifugation, the sample was immediately frozen to − 80 °C until the time of analysis. All patients were asked to respect a 12-h fast before blood collection.

### Metabolite extraction and derivatization

Metabolome extraction, purification and derivatization was carried out with the MetaboPrep GC kit (Theoreo srl, Montecorvino Pugliano [SA], Italy) according to the manufacturer’s instructions. Details regarding metabolite extraction and the overall analytical scheme, including QA/QC sample analyses, were reported in Troisi et al. [24, 66, 67].

### GC-MS analysis

Two μL samples of the derivatized solution were injected into the GC-MS system (GC-2010 Plus gas chromatograph coupled to a 2010 Plus single quadrupole mass spectrometer; Shimadzu Corp., Kyoto, Japan). Chromatographic separation was achieved with a 30 m × 0.25 mm CP-Sil 8 CB fused silica capillary GC column with 1.00 μm film thickness (Agilent, J&W Scientific, Folsom, CA, USA). High-purity helium was used as carrier gas. The initial oven temperature of 100 °C was maintained for 1 min and then raised by 4 °C/min to 320 °C with a further 4 min of hold time. The gas flow was set to obtain a constant linear velocity of 39 cm/s and the split flow was set at 1:5. The mass spectrometer was operated with electron impact ionization (70 eV) in full scan mode in the interval of 35–600 m/z with a scan velocity of 3333 amu/sec and a solvent cut time of 4.5 min. The complete GC program duration was 60 min. Untargeted metabolites were identified by comparing the mass spectrum of each chromatographic peak with the NIST library collection (NIST, Gaithersburg, MD, USA). Over 250 signals were observed in each sample, but several were not investigated further because they were either not consistently found in other sets of samples or were too low in concentration to be confirmed as metabolites due to poor spectral quality. A total of 243 endogenous metabolites involved in energy metabolism, lipid metabolism and amino acid metabolism were consistently detected and positively identified. To identify peaks, the linear index difference max tolerance was set at 10, while the minimum matching for the NIST library search was set at 85%.

#### Statistical analysis

##### Sample size evaluation

Based on the GC-MS serum metabolomic profiles of PCOS patients and controls, we have evaluated the minimum sample size to include in this pilot study to reach an 80% statistical power. Sample size was evaluated using the average power of all metabolites corrected for multiple testing using the false discovery rate (FDR = 0.20) by means of the SSPA package implemented in BioConductor [68] based on Ferreira and Zwinderman algorithms [69].

##### Anthropometric parameters

Data are reported as mean ± standard deviation for continuous variables and number (percentage) for categorical variables.

Statistical analysis was performed using Statistica software (StatSoft, Oklahoma, USA) and Minitab (Minitab Inc., Pennsylvania, USA). Normal distribution of data was verified using the Shapiro-Wilks test. Since the data were normally distributed, the t-test was used for inter-group (CTRL/PCOS-T0 and CTRL/PCOS-T1) and intra-group (PCOS-T0/PCOS-T1) comparisons. The alpha (ɑ) value was set to 0.05. Pearson’s chi-squared test was used to determine differences among groups for the categorical variables.

##### Multivariate data analysis

The chromatographic data were tabulated with one sample per row and one variable (metabolite) per column. Data pre-treatment consisted of normalizing each metabolite peak area to that of the internal standard followed by generalized log transformation and data scaling by autoscaling (mean-centered and divided by standard deviation of each variable). PLS-DA [70] was performed using the statistical software package R (Foundation for Statistical Computing, Vienna, Austria). Class separation was achieved by PLS-DA, which is a supervised method that uses multivariate regression techniques to extract, via linear combinations of original variables (X), the information that can predict class membership (Y). PLS regression was performed using the plsr function included in the R pls package [71]. Classification and cross-validation was performed using the corresponding wrapper function included in the caret package [72]. A permutation test was performed to assess the significance of class discrimination. In each permutation, a PLS-DA model was built between the data (X) and the permuted class labels (Y) using the optimal number of components determined by cross validation for the model based on the original class assignment. Two types of test statistics were used to measure class discrimination. The first is based on prediction accuracy during training. The second made use of separation distance based on the ratio *Between* group sum of the squares and the *Within* group sum of squares (B/W-ratio). If the observed test statistics was part of the distribution based on the permuted class assignments, class discrimination cannot be considered significant from a statistical point of view [73]. Variable Importance in Projection (VIP) scores were calculated for each metabolite. The VIP score is a weighted sum of squares of the PLS loadings, taking into account the amount of explained Y-variation in each dimension. The highest scoring VIP metabolites were compared in terms of fold changes (FC). FC is the ratio of the mean abundances between any two classes and is a measure describing how much a quantity changes going from an initial to a final value**.**

The metabolic pathway was constructed using MetScape application [74] of the software Cytoscape [75].

## Additional file

Additional file 1: Predicted power and sample size relationship plot. (TIFF 215 kb)

## Abbreviations

BMI: Body Mass Index
CTRL: Control Subjects
DCI: D-chiro-inositol
FC: Fold Change
FDA: Food and Drug Administration
FDR: False Discovery Rate
HOMA-IR: Homeostatic Model Assessment for Insulin Resistance
IR: Insulin Resistance
IRB: Institutional Review Board
IST: National Institute of Standard and Technology
MI: Myo-inositol
PCOS: Polycystic ovary syndrome
PCOS-T0: Polycystic ovary syndrome patients at baseline
PCOS-T1: Polycystic ovary syndrome patients after 3-months
PLS-DA: Partial Least Square Discriminant Analysis
QA/QC: Quality assurance and Quality Controls
SSPA: Sample Size and Power Analysis
TCA: Tricarboxylic Acid Cycle
VIP: Variable Importance on Projection
