# Supplementary figures and images for: Metabolomic change due to combined treatment with myo-inositol, D-chiro-inositol and glucomannan in polycystic ovarian syndrome patients: a pilot study
Source: J Ovarian Res. 2019 Mar 23;12:25. doi: 10.1186/s13048-019-0500-x (PMC6431025; doi:10.1186/s13048-019-0500-x)

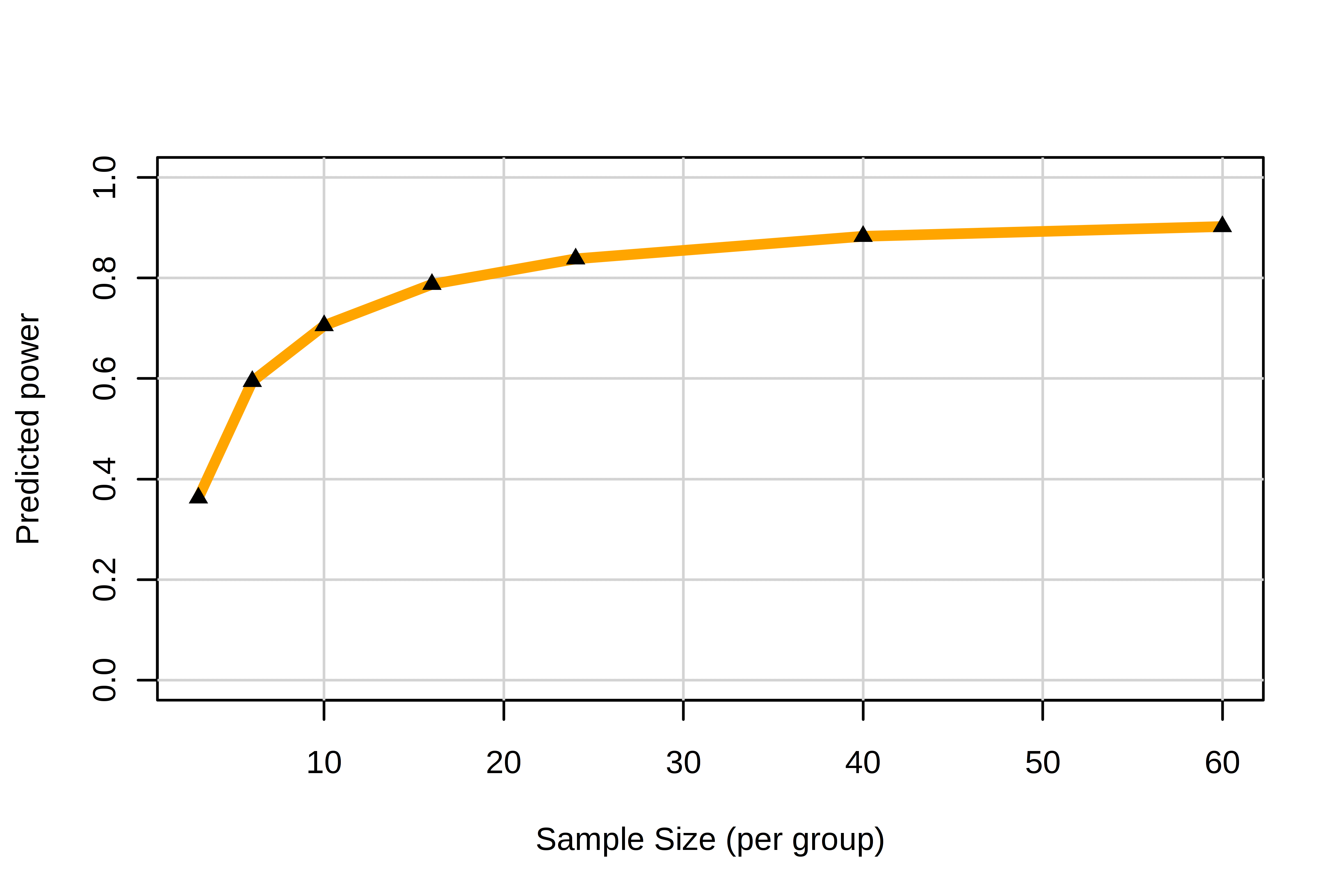

Supplement: Supplementary file 1 — Predicted power and sample size relationship plot. (TIFF 215 kb) [file 13048_2019_500_MOESM1_ESM.tiff]
